# Molecular Epidemiology of ESBL Genes and Multi-Drug Resistance in Diarrheagenic *Escherichia Coli* Strains Isolated from Adults in Iran

**Published:** 2015

**Authors:** Sadegh Ghorbani-Dalini, Mohammad Kargar, Abbas Doosti, Pejman Abbasi, Meysam Sarshar

**Affiliations:** a Jahrom Branch, Young Researcher’s & Elit Club, Islamic Azad University, Jahrom, Iran.; b Department of Microbiology, Jahrom Branch, Islamic Azad University, Jahrom, Iran.; c Biotechnology Research Center, Shahrekord Branch, Islamic Azad University, Shahrekord, Iran.; d Professor Alborzi Clinical Microbiology Research Center, Shiraz University of Medical Sciences, Shiraz, Iran.; e Department of Public Health and Infectious Diseases, Sapienza University of Rome, Rome, Italy.

**Keywords:** Antibiotic resistance, *E. coli*, ESBLs, MDR

## Abstract

Resistance to oxyimino cephalosporins antibiotics in *Enterobacteriaceae* is primarily done by the extended spectrum β-lactamases (ESBLs). Clear identification of risk factors for ESBLs-producing infections is necessary. Therefore, efficient strategies can be developed to decrease outbreak of these infections. The aim of this study was to determine the antibacterial susceptibility and ESBLs pattern of diarrhogenic *Escherichia coli* (*E. coli*) strains isolated from adult patients. In the present study, diarrheogenic *E. coli* strains were isolated from 54 patients from the University of Medical Sciences hospitals in Shiraz. Antimicrobial susceptibility testing was done by disk diffusion method by CLSI criteria. The presence of *bla*_TEM_*, bla*_SHV_ and *bla*_CTX-M_ genes was investigated by PCR using designated primers. The prevalence of ESBLs-producer *E. coli* strains was 12.96%. Antimicrobial resistance testing showed a high resistance to cefexime, trimethoprim-sulfamethoxazole, ampicillin and penicillin. Overall, β-lactamase genes were identified in 52 (96.30%) isolates which were identified as 45 (83.33%) *bla*_TEM_, 17 (31.48%) *blaSHV* and 11 (20.37%) *blaCTX*-*M*. ESBLs-producer *E. coli* is very prevalent in Diarrheogenic strains isolated from adult patients. Also, this study clearly showed that the *bla*_TEM_ gene for ESBLs-producer *E.* *coli* was widespread in Iran.

## Introduction

Diarrhea is one of the world's widespread health problems, with more than two million deaths in each year. Diarrheal disease is caused by a range of enteric pathogens such as viruses, bacteria, and parasites. Diarrheagenic *Escherichia coli *(DEC) and *Shigella *spp. are the most popular bacteria causing diarrhea (1). Emergence and dispersion of antibiotic resistance is well documented in bacterial isolates worldwide, particularly in developing countries (2, 3). Antibiotic resistance prevalence in *E. coli *is an effective marker for antibiotic resistance in each community (2, 4). *E. coli* is famous to be efficiently capable of accepting and transferring genetic materials and, under stress, readily transfers those genetic materials to enteric pathogens including *Salmonella, Yersinia*,* Vibrio, *and *Shigella *species. Therefore, it is considered an important reservoir of transferable antibiotic resistance (2-4). There is a global interest regarding advent and the rise of resistance to generally used antibiotics in bacteria (5). Irregular usage of antibiotic is possibly more important factor promoting the emergence, selection and distribution of antibiotic-resistant bacteria in human and veterinary medicine (6). This global usage of antibiotics could be connected with the selection of antibacterial resistance mechanisms in both nonpathogenic and pathogenic strains of *E. coli*. β-lactams are one of the most widely used antibiotics in both human and veterinary medicine (4). β-lactam resistance in *Enterobacteriaceae* is primarily conducted by β-lactamases. However a range of β-lactamases have been characterized, typically TEM, CTX-M and SHV enzymes are those prevalently observed among *Enterobacteriaceae* and have been increasingly found throughout the world (7, 8). Mutations in the genes encoding these enzymes can increase the spectrum of the activity of enzyme to include penicillins, the extended-spectrum cephalosporins and aztreonam. These enzymes are named extended-spectrum β-lactamases (ESBLs) (8). Antimicrobials resistant bacteria is a global problem (9). Routine monitoring of antibiotic resistance is important in order to prepare data for antibiotic therapy and resistance control (2). Also, understanding the molecular foundations of resistance can play an important role in the progress of new strategies to fight against this phenomenon (9). It is mandatory to clearly identifying the hazardous factors for infections owing to ESBL-producing bacteria, therefor efficient strategies to decrease spread of these infection agents can be developed. Various studies have been done in an attempt to identify hazardous factors for infections owing to ESBL-producer bacteria, but the results have been largely different. However these differences could be due in part to real disparity in the epidemiology of various outbreaks (10).

The aim of the present study was to determine the types of β-lactamases and antimicrobial susceptibility pattern of DEC strains recovered from diarrheal specimens of adult patients with diarrhea in the south of Iran.

## Experimental

***Samples collection***

During the period from March, 2010 to December 2010, a total of 54 DEC were collected from patients with diarrhea admitted to all of the university hospitals in Shiraz. Written informed consent was obtained from all patients. All steps of this study were approved by the Ethical Committee of Islamic Azad University. Initial isolation of samples was performed on MacConkey, EMB and VRBA and confirmed by using standard biochemical tests, including indole, TSI, citrate, urea, MR, VP, LD, OD and SIM tests (Merck, Germany).

***Phenotypic detection of ESBLs production***

A modified version of the Jarlier double-disk synergy (DDS) method (11) for detecting calvulanic acid (CLA) synergy was used. Ceftazidime (30 µg) and cefotaxime (5 µg) disks (Oxoid), were placed around an amoxicillin (20 µg)-clavulanic acid (10 µg) disk at a distance of 25 to 30 mm from center to center. A clearly visible extension of the edge of the inhibition zone of any disk towards the amoxicillinclavulanic acid disk was interpreted as positive for CLA synergy (11).

***Antimicrobial susceptibility testing***

Isolates were subjected to standard disc diffusion testing in accordance with the recommendation of the Clinical Laboratory Standard Institute (12). Isolates were tested for resistance to the following antibiotics: cefotaxime (30 μg), ceftriaxone (30 μg), cefexime (5 μg), ampicillin (10 μg), penicillin (10 U), imipenem (10 μg), ciprofloxacin (5 μg), levofloxacin (5 μg), chloramphenicol (30 μg), tetracycline (30 μg), sulfamethoxazole–trimethpprim (1.25 and 23.75 μg), gentamicin (10 μg), amikacin (30 μg) and nitrofurantoin (300 μg) (Mast Diagnostics, Merseyside, UK). Quality control was performed as recommended using the *E. coli *strain ATCC 25922.

***DNA extraction***

*E. coli *isolates were grown overnight in 5 ml of Luria Bertani (LB) broth (Merck) at 37°C. One milliliter of cell suspension for each isolate was transferred to 1.5-ml tubes and centrifuged at 13000 *rpm *for 5 min. The supernatants were removed and the cell pellets resuspended in 200 µl of sterile water by spinning. The suspensions were boiled for 15 min to lyse the cells and centrifuged as before. 150 µl of each supernatant containing DNA was removed for testing.

***PCR of β-lactamase-encoding genes***

The detection of β-lactamase-encoding genes was carried out using multiplex polymerase chain reaction (PCR) with primers that correspond to conserved regions of *bla*_TEM_ *bla*_SHV_ and *bla*_CTX-M_-type genes (Table 1). PCR amplification was performed in a reaction mixture of 25 µL**,** which contained 2 µL template DNA, 1.5 mM MgCl_2_, 0.2 mM deoxynucleoside triphosphates (dNTPs) mixture, 0.2 mM of each primer, and 1 U of *Taq *DNA polymerase (CinaGen, Co., Tehran, Iran). Initial denaturation at 95°C for 5 min; 30 cycles of 95°C for 1 min, 58°C for 1 min, and 72°C for 1 min; and, a final extension at 72°C for 5 min were used as thermal-cycling conditions. *E. coli* ATCC 35218 carrying *bla*_TEM_ and *Klebsiella pneumoniae* ATCC 700603 harboring *bla*_TEM_, *bla*_SHV_ and *bla*_CTX__-M_ were used as a positive control of amplification and *E. coli* ATCC 25922 was used as a negative control. The amplicons were visualized after electrophoresis on a 1.5% agarose gel stained with ethidium bromide.

**Table 1:** Oligonucleotide sequences used in this study.

**Primer**	**Sequence (5'→3')**	**Amplicon**	**Ref**
**TEM-F**	TCCGCTCATGAGACAATAACC	296 bp	This study
**TEM-R**	ATAATACCGCACCACATAGCAG		
**SHV-F**	TACCATGAGCGATAACAGCG	450 bp	This study
**SHC-R**	GATTTGCTGATTTCGCTCGG		
**CTX-M-F**	TCTTCCAGAATAAGGAATCCC	909 bp	(13)
**‍CTX-M-R**	CCGTTTCCGCTATTACAAAC		

## Results

***Description of sample population***

The prevalence of infected females, (53.70%) was higher than that of infected males, (46.30%). The age of patients were varied from 19 to 65 years of age. Out of the 54 *E. coli* collected, 17 (31.48%) were identified in the spring, 20 (37.04%) were in the summer, and 17 (31.48%) were in the autumn. The clinical symptoms included nausea 38 (70.37%), fever 29 (53.70%) and dysentery 7 (12.96%) (Table 2).

***Antimicrobial resistance E. coli***

The prevalence of resistance to each antimicrobial agent was: cefotaxime 16.67%, ceftriaxone 16.67%, cefexime 30.56%, imipenem 5.56%, ampicillin 36.11%, penicillin 100%, ciprofloxacin 8.33%, levofloxacin 5.56%, chloramphenicol 13.89%, tetracycline 41.67%, trimethoprim-sulfamethoxazole 41.67%, gentamycin 8.33%, amikacin 5.56% and nitrofurantoin 5.56%.

***Phenotypic results for ESBL’s***

The overall incidence of ESBLs producing isolates was 12.96% (7/54) of *E. coli* during the study period. All isolates that tested positive for ESBLs were also resistant to more than 4 antibiotics (multi-drug resistance).

***Molecular characterization of ESBL genes***

Out of 54 isolates that were subjected to PCR experiments, 52 (96.30%) of them harbored ESBLs genes. *bla*_TEM_, *bla*_SHV_ and *bla*_CTX-M_ genes were detected in 45 (83.33%), 17 (31.48%) and 11 (20.37%) of the samples, respectively. Seventeen out of 54 isolates (31.48%) carried several *bla *genes (more than one gene) while 35/54 (64.82%) of samples harbored a single *bla *gene (Table 3).

**Table 2:** Description of different criteria of patients in order to gender (n = 54).

	**Male (%)**	**Female (%)**	
18-23	8 (14.81)		5 (9.26)
24-35	8 (14.81)		13 (24.07)
36-47	3 (5.56)		7 (12.96)
48-60	5 (9.26)		1 (1.85)
>60	1 (1.85)		3 (5.56)
Spring	6 (11.11)		11 (20.37)
Summer	9 (16.67)		11 (20.37)
Fall	10 (18.52)		7 (12.96)
Nausea	17 (31.48)		21 (38.89)
Fever	13 (24.07)		16 (29.63)
Dysentery	5 (9.26)		2 (3.70)
TEM	21 (38.89)		24 (44.44)
SHV	10 (18.52)		7 (12.96)
CTX-M	3 (5.56)		8 (14.81)

**Table 3:** Distribution of ESBLs genotype of 54 diarrheagenic *E. coli*.

**Single Gene**				**Multiple Gene**				**None**
TEM	SHV	CTX-M		TEM+SHV	TEM+CTX-M	SHV+CTX-M	TEM+SHV+CTX-M	
30 (55.56)	3 (5.56)	2 (3.70)		8 (14.81)	3 (5.56)	2 (3.70)	4 (7.41)	2 (3.70)

## Discussion

Diarrheal disease owing to the different diarrheagenic *E. coli* strains is a health problem around the world, especially in developing countries. Also, it has contributed exceedingly to morbidity, mortality and increased health costs (14, 15, 16). Diarrheal diseases are rank as the fourth prevalent cause of mortality, and it has been considered that infectious diseases cause 9.2 million deaths in the developing countries (14). Antibiotic resistant diarrheagenic *E. coli *strains are usually associated with β-lactam antibiotics. In order to efficient use of antibiotics in clinical management and treatment, continuous monitoring of antimicrobial resistance is absolutely necessary (14, 17, 18, 19). Hospital transfer and antibiotic usage, particularly oxyiminocephalosporins, are well established risk factors for the distribution of ESBLs producing bacteria (18). This study prepares information regarding the problem of antibiotic resistance in diarrheagenic *E. coli *strains isolated from patients enrolled in clinics, hospitals and outpatient facility. Results showed that these *E. coli *strains have elevated rates of resistance to the currently prescribed antibiotics. Resistance against penicillins and fulate inhibitors was very high. Ampicillin resistance among *E. coli *strains was 36.11%, which is lower than the other study in Iran (14). The ampicillin resistance among diarrheagenic *E. coli *is probably due to continuous use of it for many years (20, 21). The imipenem resistance was found to be 5.56%. Imipenem is a carbapenem antibiotic, and is highly active against ESBLs producing *Enterobactereaceae*. This drug is highly resistant to beta-lactamase and has an unusual property. It causes a post antibiotic effect on Gram-negative bacteria (22). Gentamicin resistance was found to be 8.33%, which is low as compared to study reported by Aslani *et al*. (14). However, in our study, we found a 5.56% resistance to amikacin and found it be a more effective aminoglycoside against diarrheagenic *E. coli*. The resistance rate was low against aminoglycosides *i.e.*, gentamicin and amikacin, but it was equal to carbapenem, nitrofurantoines and fluoroquinolones. Resistance to ciprofloxacin was 8.33% in our study, which is in agreement with another report from Iran (14). This resistance may be owing to the use of flouroquinolones as the drug of choice in urinary tract infections (UTI) (20). The resistance rates reported in this study is in agreement with some reports from Vietnam (1), Nigeria (3), Iran (14) and Brazil (22) but are lower than Spain (7), USA (10), Thailand (18) and Pakistan (20). Many factors may have been involved with increased rates of antibiotic resistance including: mishandle of antibiotics by health care professionals or non-skilled practitioners, misuse of antibiotics by the general public (antibiotics can be used in some regions without a doctor’s prescription from pharmacy), and inadequate surveillance due to a lack of information arising from routine antimicrobial susceptibility testing, such as reports from other developing countries (20, 21, 22). Controlling the emergence and spread of ESBLs organisms involves a combination of controlling antibiotic use and strict adherence to hospital infection control measures. Restriction of one class of antibiotics can lead to increased use of another class with an accompanying increase in resistance rates. Attempts have been made to decrease the prevalence of ESBLs producing organisms by substituting earlier cephalosporins with a fourth-generation cephalosporin or beta-lactam/beta-lactamase inhibitor combinations (20). Cefotaxime and ceftriaxone resistance in *E. coli *strains described in this paper was largely associated with TEM β-lactamase genes, with only one isolate negative for TEM β-lactamase gene, but positive for both SHV and CTX-M β-lactamase genes. Although CTX-M types of ESBLs have been known for their rapid spread in many parts of the world (18, 23-27), it was remarkable that in this study, *bla*_TEM_ is more prevalent than other types of ESBLs gene in diarrheagenic *E. coli* strain. This finding is in agreement with other studies in center (14, 28, 29) and south (30-33) of Iran that showed *bla*_TEM_ is more prevalent in *E. coli*. In conclusion, *E. coli *involved in diarrhea isolated from patients enrolled in hospitals and clinics showed resistance to many antimicrobial agents, especially penicillin, ampicillin, trimethoprim-sulfamethoxazole, and cefexime, resulting in a very high percentage of resistance isolates. Also, the high number of ESBLs producing isolates gives rise to concern. This study clearly showed that the TEM β-lactamase gene, for ESBLs-producing *E. coli*, was highly endemic in Iran. Regular monitoring of resistance to antimicrobial drugs and ESBLs would seem to be necessary to improve our guidelines for empirical antibiotic therapy.
